# Unraveling the Black Box: Exploring Usage Patterns of a Blended Treatment for Depression in a Multicenter Study

**DOI:** 10.2196/12707

**Published:** 2019-07-25

**Authors:** Lise L Kemmeren, Anneke van Schaik, Johannes H Smit, Jeroen Ruwaard, Artur Rocha, Mário Henriques, David Daniel Ebert, Ingrid Titzler, Jean-Baptiste Hazo, Maya Dorsey, Katarzyna Zukowska, Heleen Riper

**Affiliations:** 1 Department of Research and Innovation GGZ inGeest Specialized Mental Health Care Amsterdam Netherlands; 2 Psychiatry, Amsterdam Public Health Research Institute Amsterdam Universitair Medische Centra, Vrije Universiteit Amsterdam Amsterdam Netherlands; 3 Centre for Information Systems and Computer Graphics Institute for Systems Engineering and Computers, Technology and Science Porto Portugal; 4 Department of Clinical Psychology and Psychotherapy Friedrich-Alexander University of Erlangen-Nürnberg Erlangen Germany; 5 Eceve, Unit 1123, Inserm Université de Paris Paris France; 6 Unité de Recherche en Economie de la Santé, Assistance Publique Hôpitaux de Paris Paris France; 7 World Health Organization Collaborating Centre for Research and Training in Mental Health Lille France; 8 Faculty of Psychology SWPS University of Social Sciences and Humanities Warsaw Poland; 9 Institute of Telepsychiatry University of Southern Denmark Odense Denmark

**Keywords:** cognitive behavior therapy, internet, combined modality therapy, depression, routine mental healthcare, treatment compliance, logfile analysis

## Abstract

**Background:**

Blended treatments, combining digital components with face-to-face (FTF) therapy, are starting to find their way into mental health care. Knowledge on how blended treatments should be set up is, however, still limited. To further explore and optimize blended treatment protocols, it is important to obtain a full picture of what actually happens during treatments when applied in routine mental health care.

**Objective:**

The aims of this study were to gain insight into the usage of the different components of a blended cognitive behavioral therapy (bCBT) for depression and reflect on actual engagement as compared with intended application, compare bCBT usage between primary and specialized care, and explore different usage patterns.

**Methods:**

Data used were collected from participants of the European Comparative Effectiveness Research on Internet-Based Depression Treatment project, a European multisite randomized controlled trial comparing bCBT with regular care for depression. Patients were recruited in primary and specialized routine mental health care settings between February 2015 and December 2017. Analyses were performed on the group of participants allocated to the bCBT condition who made use of the Moodbuster platform and for whom data from all blended components were available (n=200). Included patients were from Germany, Poland, the Netherlands, and France; 64.5% (129/200) were female and the average age was 42 years (range 18-74 years).

**Results:**

Overall, there was a large variability in the usage of the blended treatment. A clear distinction between care settings was observed, with longer treatment duration and more FTF sessions in specialized care and a more active and intensive usage of the Web-based component by the patients in primary care. Of the patients who started the bCBT, 89.5% (179/200) also continued with this treatment format. Treatment preference, educational level, and the number of comorbid disorders were associated with bCBT engagement.

**Conclusions:**

Blended treatments can be applied to a group of patients being treated for depression in routine mental health care. Rather than striving for an optimal blend, a more personalized blended care approach seems to be the most suitable. The next step is to gain more insight into the clinical and cost-effectiveness of blended treatments and to further facilitate uptake in routine mental health care.

## Introduction

Web-based interventions for depressive disorders have been studied and applied in different ways [1]. In the general population and in primary care, mainly unguided self-help and guided Web-based interventions have been evaluated [2,3]. In controlled research settings, Web-based interventions have proven to be efficacious in the treatment of depression when compared with nonintervening [4,5], even for patients with more severe symptoms [6,7]. Direct comparisons of Web-based and face-to-face (FTF) treatment formats indicated equivalence [8], and it has been shown that guided Web-based interventions lead to better treatment outcomes compared with unguided treatments [9-11]. These effects tend to be replicated in clinical practice [12-15], although effectiveness studies of Web-based interventions for depression among routine care populations are scarce and upscaling is still limited.

For the treatment of patients in routine mental health care settings, so called *blended* formats combining digital components with FTF therapy in one integrated treatment protocol are being developed, investigated, and implemented [16]. This way of working may better suit the regular practice and skills of psychotherapists and help meet the ethical and current professional guidelines [17]. As routine care mostly involves contact with a health care professional, this already implies a form of blending. This approach also constitutes a response to reported concerns of mental health care professionals about the suitability and appropriateness of Web-based treatments without FTF contact, especially for patients with moderate to severe depressive complaints [18]. Many therapists and patients express the need for an FTF interaction in these situations [19] and the desire to use internet and mobile interventions more freely integrated into FTF therapy [20,21]. Guidance from a care provider has also been identified as a key feature to improve engagement with Web-based interventions [22-24]. Thus, among more severe and complex patient groups, it may be favorable to complement Web-based interventions with FTF treatment contacts with a professional to monitor treatment progress and symptom course (eg, suicidality) and enhance motivation, compliance, and therapeutic alliance. Furthermore, a significantly higher acceptance for blended treatment, compared with stand-alone internet interventions, was shown in a survey conducted among a wide group of European mental health stakeholders involved in receiving and providing depression treatment in the adult population [25].

Preliminary findings of the few studies conducted so far suggest that blended treatments are feasible and achieve promising results in the treatment of depression [26-31]. Predominantly positive evaluations [26,28,30], high treatment satisfaction [29], and a reduction of depressive symptoms [26-28,30] were reported. Although all these studies integrated digital components with FTF therapy, the blended formats differ regarding intensity of treatment, treatment duration, the ratio of blending and degree of flexibility, and technologies employed. The FTF and Web-based sessions in, for example, the study of Kooistra et al [29] were highly structured, whereas others provided more flexibility in order and dosage for a more adaptable approach [28,30]. All treatments were based on cognitive behavioral therapy (CBT) elements. In most studies so far, FTF sessions were combined with the use of a Web-based platform. In the blended treatment by Ly et al [27], however, a mobile phone platform, instead of a Web-based platform, was included as the digital component. The use of smartphones also facilitates the integration of ecological momentary assessment (EMA)—the real-time monitoring of contextual variables experienced in a daily life context [32]—to improve the assessment of mood and behaviors and understand their relationship [33]. Besides a diversity in the type of technology, a variety of Web-based platforms are being used. In the large-scale European study (*European Comparative Effectiveness Research on Internet-Based Depression Treatment*, ie, E-COMPARED), which was recently completed, a blended treatment protocol combining FTF sessions with Web-based, as well as mobile, elements was used [34].

Although there are many possible applications of blended treatment in mental health care, knowledge on how blended treatments should be set up optimally is still limited [35]. Blended treatments have primarily been evaluated as a treatment package, not taking into account how the individual elements, such as FTF sessions or Web-based modules, contributed to the results. The effectiveness of this form of treatment remains hard to determine because of the lack of research into the different intervention characteristics. In addition, the application of an intervention as it is designed—also referred to as treatment fidelity [36]—has important implications for the interpretation of treatment outcomes. Lack of attention to treatment fidelity increases the risk of the inability to draw solid conclusions, as treatment effects may be attributed to other factors unrelated to the treatment itself [37]. To further explore and optimize blended treatment strategies, it is thus important to obtain a full picture of what actually happens during the treatment, taking all blended components into account separately: the usage of digital elements by the patient, the Web-based feedback provided by the therapist, and the real-time contact that has taken place between patient and therapist. As the use of internet modules and mobile apps can systematically be logged, objective and detailed measures of intervention use are available. With these log data, broad and in-depth information on how patients use and proceed through the intervention can be provided along with a realistic estimation of exposure to intervention content [38]. Combining log data with self-report questionnaire data provides a rich source of information describing the usage of the different components of blended treatment protocols in routine mental health care.

In this study, set out in the context of the E-COMPARED project, we intended to unravel the different elements of a blended depression treatment for adults. The first aim of the study was to describe the usage of blended Cognitive Behavioral Therapy (bCBT), looking at all its constituent components—FTF and Web-based contact between patient and therapist and the use of Web environment and mobile app. This was conducted across 4 European countries participating in the E-COMPARED trial and using the same digital platform (*Moodbuster*) with which they provided the Web-based modules for the bCBT. The second aim was to reflect on the actual usage of the blended treatment in routine practice, as compared with the intended application of the blended treatment protocol in each country. The third aim was to compare differences in usage patterns of bCBT between primary (Germany and Poland) and specialized care settings (the Netherlands and France), and the fourth was to identify who complies with a blended treatment approach, based on usage intensity and integration of the FTF and Web-based components. This will contribute to a better understanding of which patients do (or do not) engage with the bCBT and what that engagement looks like.

## Methods

### Design

Data were extracted from the research database of the E-COMPARED project [39]. This study was a pragmatic, multinational, randomized controlled trial in 9 European countries (N=943) and aimed to compare the effectiveness of blended treatment for major depression with that of treatment-as-usual (TAU) [34]. The blended treatment was provided across different sites in primary or specialized mental health care services. Various Web-based platforms were used, depending on the availability of existing systems and specific needs of the participating country. Due to the technical abilities of the Moodbuster platform to log treatment use, the focus of this paper is on the 4 countries that specifically used this platform for the blended intervention, namely, Germany, the Netherlands, France, and Poland. The Moodbuster platform was also used in the United Kingdom, however, parallel to another messaging system. As the Web-based communication could not be retrieved, participants from the United Kingdom were not included in this study because of this missing component.

### Participants

Recruitment took place in routine mental health care settings, between February 2015 and December 2017. Germany and Poland recruited patients in primary care (general practices and primary care centers) and France and the Netherlands recruited in specialized mental health care settings (outpatient clinics). Patients (aged 18 years and older) with a primary diagnosis of major depressive disorder, who were indicated for depression treatment, were asked by their health care professional if they were willing to participate in the study. If so, they were contacted by a research assistant who screened them for eligibility. Depressive disorder had to be confirmed by the Mini International Neuropsychiatric Interview (MINI) and a score of ≥5 on the Patient Health Questionnaire-9 (PHQ-9). Patients should not be receiving other psychological treatment for depression. However, there were no restrictions regarding medication use. After inclusion, patients were randomized to bCBT or TAU. TAU was the routine depression care offered in the specific treatment setting where patients were recruited and could comprise psychotherapy, pharmacotherapy, or a combination of both. Patients were followed up at 3, 6, and 12 months after baseline. Neither therapists nor patients were compensated for study participation. Further information on recruitment procedures and inclusion and exclusion criteria has been specified elsewhere [34]. The recruitment process resulted in 231 participants who were randomized to the Moodbuster blended intervention. Of these, 31 did not start the allocated intervention or had no data available on FTF contacts. Therefore, bCBT data of in total 200 participants could be included in this paper.

### Blended Intervention

**Figure 1:**
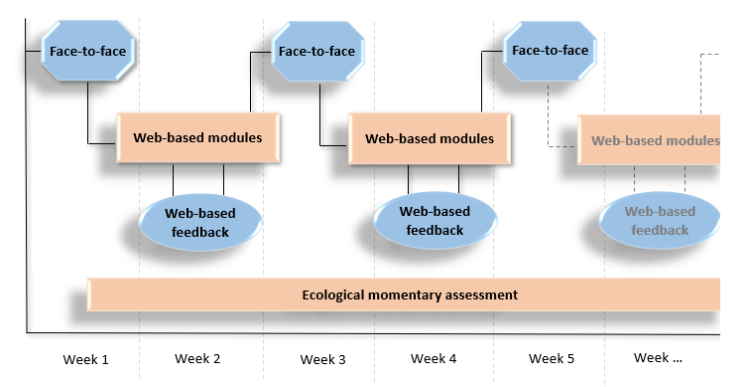
The 4 blended elements presented over time: blended cognitive behavioral therapy always starts with a 'face-to-face' session. Patients work on the Moodbuster 'Web-based modules' in between the face-to-face sessions and receive 'Web-based feedback' from their therapist through a written message on Moodbuster. During the entire treatment period, patients track their mood daily on the mobile application ('Ecological momentary assessment’).

The blended depression treatment in this study integrates individual FTF therapy with both internet- and mobile-based interventions. FTF CBT sessions are alternated with Web-based sessions, delivered through an internet-based treatment platform called Moodbuster [40]. Moodbuster is a research platform initially developed within the ICT4Depression project [41,42] and adapted for the blended intervention within the E-COMPARED project [43]. While completing the Web-based modules in between the FTF sessions, patients receive Web-based support from their therapist in the form of a personalized written feedback message. In addition to that, patients make use of a mobile app for depression symptom monitoring and other contextual variables such as sleep, rumination, and social interactions [44]. Figure 1 illustrates the administration of the different blended components over the course of the intervention. First, we elaborate on the application of the blended treatment protocol as a whole across participating countries. Subsequently, the elements of the blended treatment are further outlined.

#### Blended Treatment Protocol

The blended treatment was provided across different European countries, with a diversity in settings and health care infrastructures. A generic bCBT protocol was set up, permitting treatment to be tailored to the local sites and situations but at the same time, preventing too much heterogeneity between blended treatments [34]. Similar bCBT was provided in all countries, including the same CBT core elements, and on the same Web-based treatment platform. The number of sessions and the ratio between FTF and Web-based sessions could vary according to local practice, but within a given range: a minimum of one-third of the sessions had to be FTF and a minimum of one-third Web-based. Also, planned treatment duration could differ across research sites. This variation was enabled to increase fit with local health care infrastructure, as the blended treatment was provided in routine care settings. The treatment duration and the ratio of FTF and Web-based sessions (see Table 1) determined the intensity of the treatment. In Germany and Poland, treatment was delivered to patients who were recruited in primary care, in a scheduled treatment duration of 7 to 13 weeks. In France and the Netherlands, patients were recruited in specialized treatment settings and the scheduled treatment duration was 16 to 20 weeks.

**Figure 2:**
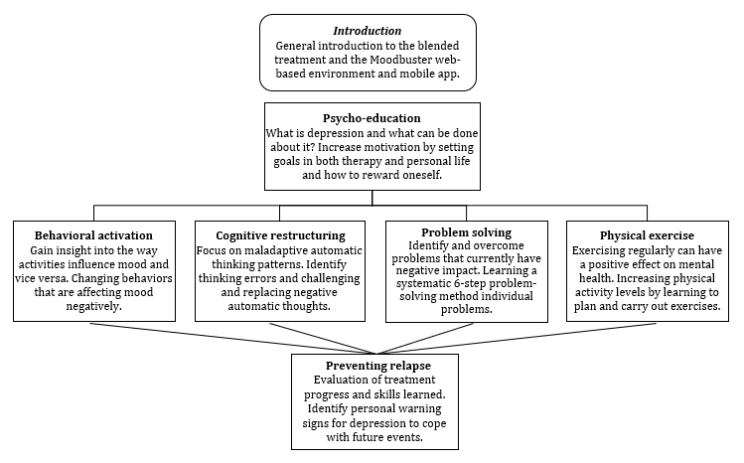
Moodbuster module flowchart of the blended treatment protocol in E-COMPARED (European Comparative Effectiveness Research on Internet-Based Depression Treatment).

The blended treatment always started with an FTF meeting in which the blended format was discussed. After that, 2 mandatory Web-based modules *Introduction* and *Psychoeducation* were to be completed by patients. After completion of these 2 modules, automatic access was granted to the remaining therapeutic modules (except *Relapse Prevention*). These modules, namely, *Behavioral Activation*, *Cognitive Restructuring*, *Problem Solving*, and *Physical Exercise*, could be followed in any preferred sequence based on patient’s preferences and therapist’s assessment. Patients were, however, requested to complete, with guidance from the therapists, at least the modules *Cognitive Restructuring* and *Behavioral Activation* during the treatment *,* as these were parts of the core components of CBT. *Problem Solving* and *Physical Exercise* were seen as optional. Across countries, all treatments were intended to end with the *Relapse Prevention* module, for which therapists granted access attuned to individual patient time frames. The module flowchart is illustrated in Figure 2.

Patients were instructed to work on 1 module at a time. Moodbuster allows for differences in paths and tempo when proceeding through the intervention. The introduction pages of the modules were accessible at all times, but before entering a new module the patient had to confirm that the choice to activate that module was made in agreement with the therapist. The recommended time frame to progress through a module was 1 to 2 weeks, but more time was given if needed. Overall, flexibility was given for sequence and time spent on each module. End of treatment was defined as *last FTF* or *last Web-based contact with patient*.

**Table 1:** Scheduled blended treatment format per country in the E-COMPARED (European Comparative Effectiveness Research on Internet-Based Depression Treatment) trial.

Country	Type of care	Treatment duration (weeks)	Face-to-face/Web-based ratio^a^
Germany	Primary	11-13	6/10
Poland	Primary	7-10	7/6
The Netherlands	Secondary	18-20	10/9
France	Secondary	16-20	8/8

^a^Face-to-face/Web-based ratio represents the total number of recommended face-to-face and Web-based sessions.

#### Blended Treatment Components

##### Face-to-Face Contact With Therapist

The FTF sessions were provided by therapists who were trained specifically in the blended CBT format with Moodbuster. During training, therapists were instructed on the content of the Web-based modules, how to combine FTF treatment with Web-based modules, how to structure sessions, how to use the Moodbuster platform, and how to deliver the Web-based feedback to patients. At all sites, therapists were provided with a treatment manual describing therapy sessions; regular supervision meetings were conducted; and ongoing support was available when necessary. In all countries, therapists were either licensed psychotherapists with experience in CBT or CBT therapists in training (with a university degree in psychology) who worked under the supervision of an experienced psychotherapist.

The FTF sessions followed up on the Moodbuster modules and were used to discuss content and exercises in more depth. The task of the therapist was to motivate and increase adherence to Moodbuster, as well as to guide patients through the modules and personalize the therapy. Instructions on how to structure the FTF sessions included, for example, reflecting on mood ratings, reviewing the last edited treatment module, repeating or clarifying exercises and lessons learned from the Web-based module, deepening personal therapy themes, and deciding on and discussing the next Web-based module.

##### Web-Based Treatment Modules for Patients

Patients were given access to the Moodbuster treatment platform with a secure personal login. Within the patient Web portal, access is given to the treatment modules, homework exercises, mood graph, calendar, and messaging system. Moodbuster is currently available in 6 languages: English, Dutch, German, Polish, French, and Portuguese.

Moodbuster comprises 1 introduction module and 6 interactive treatment modules targeting depression (see Figure 2). Each therapeutic module focusses on a specific evidence-based psychotherapeutic element, such as cognitive restructuring. Modules comprise a series of pages that patients must complete in sequence. All modules start with a description of the goal and content of the therapeutic approach, followed by an illustrative video. Module pages contain either reading material combined with illustrations, examples, and tips as a didactical part or an interactive homework exercise where patients can apply the offered information to their own situations. All modules end with a summary followed by the administration of a questionnaire, evaluating the module and assessing severity of depressive symptoms. Screenshots of the Moodbuster website can be found in Multimedia Appendix 1.

##### Web-Based Feedback From Therapist

Therapists provided asynchronous Web-based feedback through the secure Moodbuster message system on a prescheduled time point in between the FTF sessions. Through the therapist Web portal, therapists were able to track their patients’ progress and homework exercises on the website, as well as their mood registrations from the mobile phone app (see Multimedia Appendix 1). This information was used to provide written feedback. The goal of the Web-based feedback was to support the patient in the uptake of the content, encourage reflection, and motivate the usage of the Web-based modules. Therapists were provided with guidelines and examples for feedback messages, but specific content was adapted based on the progress of the patient.

##### Mobile App

Patients were prompted daily on their smartphone to rate their mood on a 1 to 10 visual analogue scale. At a random time point between 10 am and 10 pm, patients were presented with the following question: “How is your mood right now?” There was a 60-min time frame to respond to the prompt. In addition, patients could also register their mood at a self-appointed time. Besides the daily monitoring of mood, patients were also presented with questions on their sleep, activity level, social interaction, self-esteem, and rumination [44]. The monitoring of mood symptoms was presented in an interactive graph that was viewable for both patients and therapists on the website and the mobile app (see Multimedia Appendix 1). The mood graph was intended to support treatment progression and active participation of the patients. In this paper, we only included the mood registrations, as only these were used as a clinical support tool in the blended treatment.

### Measurements

#### Patient Characteristics

Demographic variables, including gender, age, partner status, and educational level, were collected through a Web-based questionnaire at baseline. Depression severity was assessed with the PHQ-9 [45]. Patients score each of the 9 Diagnostic and Statistical Manual of Mental Disorders (4th edition) criteria on a 4-point scale, ranging from 0 (not at all) to 3 (nearly every day). The total score ranges from 0 to 27, with higher scores indicating greater symptom severity. The PHQ-9 has shown good psychometrics, with Cronbach alpha of .89 [45,46].

To assess a diagnosis of lifetime and current depression and current comorbid psychiatric disorders, the MINI [47] version 5.0 was conducted at baseline. Before being allocated to 1 of the 2 conditions, patients were asked to indicate their treatment preference (*bCBT*, *TAU*, or *no preference*) within the Web-based questionnaire.

#### Treatment Elements of Blended Cognitive Behavioral Therapy

##### Face-to-Face Contacts

To evaluate treatment fidelity, all therapists were asked to register the date, duration of the contact, and the main (module) interventions discussed after each FTF session with a patient. From this, the number and frequency of FTF sessions within the blended intervention were derived. This checklist was developed and applied within the E-COMPARED project to assess treatment exposure in both groups (TAU vs bCBT) and make it possible to compare these.

##### Moodbuster Website and Mobile Use (Log Files)

In the Moodbuster system, activities on the platform were systematically collected. The resulting log files contained detailed logs of system usage, including for how long the system was used, how many times the website was visited within this period, the amount of time spent on the website, patients’ interaction with the Moodbuster module Web pages, and messages exchanged between the patient and the therapist. Moodbuster automatically logged when patients opened and closed module pages. This information was used to calculate frequency, duration, order, and completion of the Web-based modules, providing insight into usage patterns. As users may be interrupted within a session and leave a module page open without formally logging out, the Moodbuster system would automatically log out if a patient was inactive for >30 min. In this way, overestimation of total duration on the Moodbuster website was limited. The mobile measures were date- and timestamped, providing information on the number of mood registrations and usage weeks. Moodbuster usage data were assessed over the course of 6 months, spanning 26 weeks after the first login. Table 2 gives a full overview of the usage metrics that were extracted from the log files.

**Table 2:** Moodbuster usage metrics, as extracted from raw logfile data.

Usage metrics		Description
**Website**		
	N^a^ of usage weeks	Total number of weeks that Moodbuster has been used by the patient, based on the first and the last login date
	N of logins	Total number of times that the patient logged on to the Moodbuster website
	N of modules started	Total number of modules where the patient reached at least page 3 (confirming having started the module)
	N of modules completed	Total number of modules where the patient visited all pages and filled in the end-of-module questionnaire
	Average login duration	Average number of minutes spent on the Moodbuster website per login
	Total usage duration	Total minutes spent on the Moodbuster website
	N of messages	Total number of messages sent by the therapist or patient
	Message length	Average number of characters used per message from the therapist or patient
	N of contact weeks	Total number of weeks between the first and the last Web-based message from the therapist or patient
**Mobile app**		
	N of mood registrations	Total number of times that patient registered mood state on the Moodbuster mobile app

^a^N: total number.

### Analysis

For the analyses, diagnostic interview and questionnaire data were merged with the log files of the Moodbuster platform (website and mood response rates). Patient characteristics and usage of the different blended treatment components were analyzed using descriptive statistics. To assess for differences in demographics and baseline scores among the 4 countries, between mental health care settings, and between the patients being compliant and noncompliant with the bCBT format (based on engagement with FTF and Web-based components), independent samples *t* test and 1-way analysis of variance for continuous variables and chi-square tests for categorical variables were conducted. Post hoc tests (Tukey honest significant difference) were run to confirm where significant differences occurred among the countries. The statistical analyses were performed using IBM SPSS statistics version 24.0 [48]. Statistical significance was set at *P*<.05 (2-sided). Due to the exploratory nature of this study, we did not correct for multiple testing.

### Ethical Approval

Ethical approval for the trials was provided at national level (Germany: Ethik Kommison DGPsychologie, Universität Trier, MB 102014; Poland: Komisja ds. Etyki Badan Naukowych, 10/2014; The Netherlands: METC VUMC, 2015.078; France: Comité de protection des personnes, Île de France V 15033-n° 2015-A00565-44) and each trial was registered in a local clinical trial register (Germany: German Clinical Trials Register DRKS00006866; Poland: ClinicalTrials.Gov NCT02389660; the Netherlands: Netherlands Trials Register NTR4962; France: ClinicalTrials.gov NCT02542891). Written informed consent was obtained from all the participants, including permission to share anonymized data across the participating E-COMPARED partners.

## Results

### Overview

**Figure 3:**
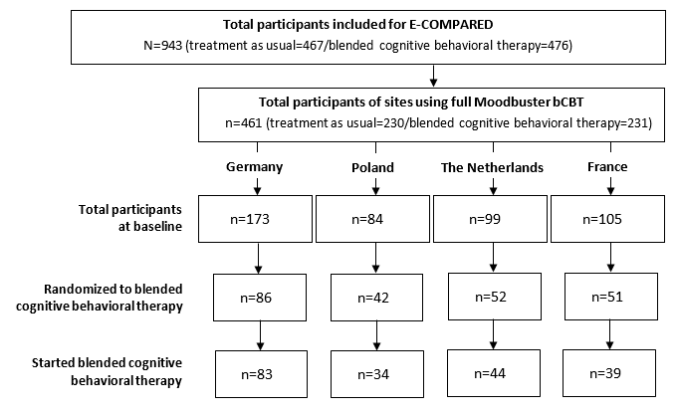
Flowchart of participants. Started blended cognitive behavioral therapy means at least 1 face-to-face session and at least 1 login on the Moodbuster platform. E-COMPARED: European Comparative Effectiveness Research on Internet-Based Depression Treatment. bCBT: blended cognitive behavioral therapy.

Of the 231 patients randomized to the Moodbuster blended intervention, 31 (31/231, 13.4%) did not receive the allocated intervention (shown in Figure 3). Of these, 29 never attended the first scheduled treatment session or dropped out after the first FTF session and never logged in to the Moodbuster website. For the 2 patients who did use Moodbuster, there were no data available on FTF contacts. There were no significant differences in demographic or clinical characteristics between the group that did and did not start bCBT.

The patient and treatment characteristics in Tables 3 and 4 are of the 200 patients for whom data on the 4 blended components were available and who started with the allocated bCBT treatment, defined as having at least 1 FTF session and at least 1 login on the Moodbuster platform.

### Patient Characteristics

Table 3 summarizes the baseline demographics and clinical characteristics of the included total sample and of each of the 4 countries separately.

The average age of the total sample was 42 years (range 18-74 years), 64.5% (129/200) were female, 51% (102/200) received higher education (postsecondary), and 58.5% (117/200) were married or were living with a partner. Overall scores on the PHQ-9 indicated moderately severe levels of depressive symptoms (PHQ>14). The number of comorbid disorders ranged from 0 to 6, including multiple anxiety disorders and substance use disorder. In total, 56.5% (113/200) had at least 1 comorbid disorder.

#### Baseline Differences

We looked at differences in demographics and clinical characteristics at baseline among the countries and the care settings. No significant differences among the countries or the care settings were found in patients’ gender or partner status. The mean age of patients significantly differed among sites, with on average younger patients in Poland (36.4 years), compared with Germany (43.2 years) and France (45.9 years) (*F*_3,196_=4.34; *P*=.005), but did not significantly differ between care settings (*F*_3,198_=0.45; *P*=.51). Educational level was differently spread across the countries (X^2^_6_=22.6;  *P*=.004), as well as between care settings (X^2^_2_=11.4;  *P*=.003). In Germany, Poland, and France, more than half of the patients (55.5%, 61.8%, and 51.3%, respectively) received higher education, whereas in the Netherlands, only 32.8% had a postsecondary education and most patients (54.5%) had a middle education level (secondary education). No significant differences were found in baseline depression severity scores among the countries or the care settings. However, a significantly higher percentage of patients in specialized care (74.7%), compared with primary care (43.6%), had one or more comorbid disorders (X^2^_1_=19.1;  *P*<.001), and the mean number of comorbid disorders in Germany (0.6) and Poland (0.9) was significantly lower than that in the Netherlands (1.5) and France (1.7) (*F*_3,186_=15.35; *P*<.001). Assessed treatment modality preferences at baseline significantly differed among the countries and the care settings. In Germany and Poland (primary care), the majority of the patients expressed a preference for blended treatment (77.1% and 70.6%, respectively; primary care: 75.2%), as opposed to only 20.5% in France and 47.7% in the Netherlands (specialized care 34.9%) (X^2^_6_=46.1;  *P*<.001). In specialized care, a significantly higher percentage expressed a preference for TAU (26.5%), as compared with primary care (6.0%) (X^2^_2_=34.5;  *P*<.001).

**Table 3:** Baseline patient characteristics (N=200).

Patient characteristics		Total	Primary care		Specialized care		*P* value
			DE^a^ (n=83)	PL^b^ (n=34)	NL^c^ (n=44)	FR^d^ (n=39)	
Gender (female), n (%)		129 (64.5)	51 (61.4)	25 (73.5)	27 (61.4)	26 (66.7)	.61
Age (years), mean (SD)		41.7 (12.9)	43.2 (13.1)	36.4 (13.1)	39.4 (9.8)	45.9 (13.6)	.005
**Education level, n** **(%)**							
	Low	24 (12.0)	15 (18.1)	2 (5.9)	6 (13.6)	1 (2.6)	.004
	Middle	74 (37.0)	21 (25.3)	11 (32.4)	24 (54.5)	18 (46.2)	.004
	High	102 (51.0)	47 (56.6)	21 (61.8)	14 (31.8)	20 (51.3)	.004
In a relationship, n (%)		117 (58.5)	51 (61.4)	24 (70.6)	24 (54.5)	18 (46.2)	.17
Baseline PHQ^e^, mean (SD)		16.2 (4.7)	15.5 (4.1)	16.3 (5.0)	16.9 (5.8)	16.8 (4.6)	.33
Comorbidity, n (%)^f^		113 (60.1)	34 (41.5)	17 (50.0.9)	34 (77.3)	28 (71.8)	<.001
**Treatment preference, n** **(%)**							
	Blended	117 (58.5)	64 (77.1)	24 (70.6)	21 (47.7)	8 (20.5)	<.001
	TAU^g^	29 (14.5)	6 (7.2)	1 (3.3)	7 (15.9)	15 (38.5)	<.001
	Non^h^	54 (27.0)	13 (15.7)	9 (26.5)	16 (36.4)	16 (41.0)	<.001

^a^DE: Germany.

^b^PL: Poland.

^c^NL: the Netherlands.

^d^FR: France.

^e^PHQ: Patient Health Questionnaire-9.

^f^≥1 comorbid disorder, as assessed with the Mini International Neuropsychiatric Interview.

^g^TAU: treatment-as-usual.

^h^Non: no treatment preference.

### Usage of Blended Cognitive Behavioral Therapy

The detailed usage of the blended treatment components per country is presented in Table 4. In general, patients demonstrated a large variability in usage of the blended treatment. The observed patterns of treatment duration and ratio between FTF and Web-based sessions to some extent correspond with the intended application. Looking at the number of FTF sessions, we overall tend to see fewer differences between sites than may have been expected based on the variances in the scheduled amounts. First, we describe and reflect on intended versus observed application of the bCBT in each country (see scheduled treatment duration and session frequency in Table 1). Furthermore, we compare the application of bCBT between primary and specialized care. Next, we look at general patterns in engagement with bCBT, based on the integration of FTF and Web-based components in the treatment process. Finally, we test for differences in patient characteristics between the blended and nonblended compliant group.

**Table 4:** Usage of blended treatment elements per country.

Blended treatment elements			Primary care		Specialized care	
			DE^a^ (n=83)	PL^b^ (n=34)	NL^c^ (n=44)	FR^d^ (n=39)
**Face-to-face sessions**						
	**Total number of face-to-face sessions**					
		Mean (SD)	4.9 (0.6)	6.7 (3.5)	7.5 (5.0)	7.4 (2.3)
		Range	2-5	1-18	2-32	2-9
	**Total duration of face-to-face contacts in minutes**					
		Mean (SD)	275 (42)	342 (182)	346 (230)	427 (146)
		Range	105-387	60-925	90-1440	100-595
	**Average duration of face-to-face contact in minutes**					
		Mean (SD)	54.9 (8.3)	52.0 (7.3)	46.2 (5.8)	56.8 (6.7)
		Range	21-77	30-70	33-64	31-67
	**Number of contact weeks**					
		Mean (SD)	9.4 (3.2)	9.5 (6.5)	16.5 (11.2)	15.8 (6.1)
		Range	2-26.1	0-30.3	2-61	2-28
**Moodbuster website usage**						
	**Number of usage weeks**					
		Mean (SD)	12.6 (3.8)	10.6 (5.6)	13.2 (7.0)	13.9 (6.5)
		Range	2.2-24.8	0.7-25.3	1.9-25.9	0-26.5
	**Number of logins**					
		Mean (SD)	16.7 (7.1)	15.8 (7.9)	11.9 (9.2)	15.5 (13.9)
		Range	4-34	2-39	3-40	1-66
	**Number of modules started**					
		Mean (SD)	6.6 (1.0)	6.1 (1.3)	4.5 (1.5)	5.0 (2.0)
		Range	3-7	3-7	2-7	1-7
	**Number of modules completed**					
		Mean (SD)	6.4 (1.3)	5.6 (1.7)	3.8 (1.6)	4.4 (2.1)
		Range	2-7	2-7	2-7	1-7
	**Total usage duration in minutes**					
		Mean (SD)	368 (193)	396 (246)	228 (193)	332 (333)
		Range	86-1199	54-1087	53-876	22-1455
**Moodbuster Web-based messages usage**						
	**Number of messages from therapist**					
		Mean (SD)	15.3 (5.9)	6.0 (3.2)	5.8 (3.9)	4.3 (3.7)
		Range	4-39	0-13	0-14	0-12
	**Number of messages from patient**					
		Mean (SD)	8.3 (5.5)	3.8 (2.4)	4.6 (4.0)	3.1 (2.9)
		Range	2-32	0-11	0-17	0-12
	**Average message length from therapist (number of characters)**					
		Mean (SD)	1282 (353)	471 (284)	741 (372)	432 (148)
		Range	613-2316	140-1026	133-1636	120-702


	**Average message length from patient (number of characters)**					
		Mean (SD)	450 (420)	421 (373)	533 (350)	293 (125)
		Range	137-3874	115-1475	132-1522	94-529
	**Number of contact weeks**					
		Mean (SD)	11.9 (3.6)	7.3 (4.7)	9.7 (7.2)	8.1 (6.0)
		Range	1-23.9	0-18.1	0-24.9	0-18
**Moodbuster mobile app usage**						
	**Number of usage weeks**					
		Mean (SD)	14 (5.8)	12.3 (7.9)	14.7 (8.5)	14 (6.5)
		Range	2.9-25.9	0-25.8	0-25.7	0-25.5
	**Number of mood registrations**					
		Mean (SD)	128.2 (92)	58 (42.8)	71.5 (72.8)	68.1 (38.5)
		Range	16-442	3-204	1-352	1-158

^a^DE: Germany.

^b^PL: Poland.

^c^NL: the Netherlands.

^d^FR: France.

#### Germany

In Germany, 94% (78/83) of patients attended 5 FTF sessions (mean 4.9, SD 0.6), provided over a period of 9.4 (SD 3.2) weeks and paired with an average usage duration of the Moodbuster website of 12.6 weeks (SD 3.8). This is in accordance with the scheduled treatment duration of 11 to 13 weeks. It should, however, be noted that the first FTF session was not registered by therapists, as it was a technical introduction and did not include therapeutic content. Thus, in practice, the prescribed number of 6 FTF sessions was also followed. The total time spent on the website averaged 6.2 hours, with a mean usage duration of 22 min per login. All patients completed the 2 mandatory modules (Introduction and Psychoeducation) and started at least 1 optional module. The number of messages sent by therapists (mean 15.5, SD 5.9) and the length of these messages (mean 1282, SD 353) was notably higher compared with the other 3 sites. This corresponds to the scheduled ratio between FTF and Web-based session, where the Web-based part had a larger share (6 FTF/10 Web-based). All patients made use of the mobile phone app, for on average 14 weeks, registering their mood on average 128 times (range 16-442). This number of mood ratings was significantly higher than that in the other countries, as well as the expected number of ratings based on daily prompts (14 weeks×7 days=98 ratings), but can be explained by the option on the Moodbuster app permitting registration of mood at any moment without being prompted.

#### Poland

Patients in Poland, on average, attended 6.7 (SD 3.5, range 0-18) FTF sessions over a time period of 9.5 (SD 6.5) weeks, which is in line with the intended application (7 FTF sessions, 7-10 weeks). A patient only attended 1 FTF session (contact weeks=0) and the longest interval between the first and the last FTF session was 30.3 weeks. The Moodbuster website was used for 10.6 weeks on average (SD 5.6), with patients spending on average a total of 6 hours and 36 min on the Web. Patients received on average 6 (SD 3.2) feedback messages over a period of 7.3 (SD 4.7) weeks, which can be seen as matching the scheduled ratio of 7 FTF and 6 Web-based sessions. A total of 2 patients (2/34, 5.9%) never exchanged any Web-based message with their therapist. The mobile app was used by 91.2% (31/34) of Polish patients, with an average of 58 mood ratings (SD 42.8). On the basis of daily prompts and an average mobile usage duration of 12.3 weeks, the number of mood ratings was lower than that would have been expected (12.3×7=86 ratings).

#### The Netherlands

The number of FTF sessions in the Netherlands averaged 7.5 (SD 5), fewer than the prescheduled average of 10 FTF sessions and with a wide range of 2 to 32 sessions. Treatment duration was on average 16.5 weeks (SD 11.2, range 2-61) and website usage duration on average 13.2 weeks (SD 7, range 1.9-25.6). Compared with the other sites, patients in the Netherlands spent less time on the Moodbuster website, with an average of 11.9 logins and a total of 3 hours and 48 min on the Web. Therapists sent a mean of 5.8 messages to their patient (range 0-13) and on average received 3.8 messages from a patient (range 0-11). This was lower than that would be expected based on the scheduled ratio (10 FTF/9 Web-based). In 3 cases (6.8%), there was never any Web-based message exchange. A total of 6 patients (6/44, 13.6%) did not use the mobile app and 3 (3/44, 6.8%) only registered their mood once. The average number of mood registrations was 71.5 (SD 72.8), less than the expected 103 mood ratings based on the average mobile usage duration of 14.7 weeks.

#### France

In France, patients had on average 7.4 (SD 2.3, range 2-9) FTF sessions with their therapist over an average period of 15.8 weeks (SD 6.1, range 2-28). This is close to the scheduled number of 8 FTF sessions and treatment duration of 16 to 20 weeks. The total time spent on the Moodbuster website averaged 5 hours and 32 min, with a wide range of 22 to 1455 min. The ratio between FTF and Web-based sessions was intended to be 50/50. Patients, however, spent less time on the Web than FTF (332 vs 427 min) and received on average 4.3 (SD 3.7) Web-based messages from their therapist with only an average of 8.1 weeks between the first and the last exchanged Web-based message. A total of 7 therapist-patient pairs (7/39, 18%) did not exchange any Web-based message. The mobile app was used by 82% (32/39) of French patients. These patients used the mobile phone for an average of 14 weeks, registering their mood on average 68.1 times (SD 38.5), which is less than the expected 98 ratings based on daily prompts.

#### Application of Blended Cognitive Behavioral Therapy in Primary and Specialized Care

There is a clear distinction visible between primary care (Germany and Poland) and specialized care (the Netherlands and France) regarding duration and intensity in the application of the different blended components. The number of contact weeks with a therapist in primary care was significantly lower than that in specialized care (on average 9.5 weeks and 16.2 weeks, respectively; *t*_198_=−6.93; *P*<.001). As intended, in primary care settings also, significantly fewer FTF sessions were provided than in specialized care (5.4 vs 7.5 FTF sessions; *t*_198_=−4.8; *P*<.001). Patients in primary care, on the other hand, started and also completed significantly more Web-based modules compared with patients in specialized care settings (6.4 vs 4.8 modules started; *t*_198_=8.26; *P*<.001 and 6.1 vs 4.1 modules completed; *t*_198_=8 .71; *P*<.001). Also, more time, on average, was spent on the Moodbuster website in primary care (376 min) compared with specialized care (277 min) (*t*_198_=2.92; *P*=.004). The therapists in primary care sent on average 12.6 messages, significantly more than the average of 5.1 messages sent by the therapists in specialized care (*t*_198_=9.12; *P*<.001). Looking at the usage of the mobile app, no differences were found in number of usage weeks. Primary care patients, however, more often rated their mood with an average of 109 ratings, compared with 70 mood ratings in specialized care (*t*_182_=3.31; *P*<.001).

#### General Impression of Engagement With Blended Cognitive Behavioral Therapy Components

To explore differences in patterns of engagement with the blended treatment, we looked at both the FTF and the Web-based parts of bCBT. For the FTF component, we focused on the total number of sessions (≥ or <3 FTF sessions), and for the Web-based part we summarized Moodbuster usage intensity by taking into account the number of Web-based modules started (≥ or <3 modules) and total login duration (≥ or <60 min). In total, 4 groups could be identified: *early dropouts*, *mainly Web-based*, *mainly FTF,* and *blended compliant*.

##### Early Dropouts

Out of 200 patients, 3 (3/200, 1.5%) received <3 FTF sessions and did not complete >2 mandatory modules (Introduction and Psychoeducation) or spent <1 hour on the Moodbuster website. These patients were considered to be early treatment dropouts.

##### Mainly Web-Based

A total of 8 patients (8/200, 4%) started at least 1 optional module (3 or more modules in total) by spending at least an hour on the Web on Moodbuster but had no more than 2 FTF sessions with their therapist. The average number of FTF sessions was 1.5 (SD 0.5, range 1-2), provided over a period of a maximum of 2 weeks (mean 1.0, SD 1.1) and paired with on average 5.2 (SD 2.3, range 2-9) Web-based messages from therapist. Patients started 4.6 (SD 1.3) and completed 3.8 (SD 1.4) Web-based modules on average. The mean number of logins was 9.4 (SD 3, range 4-12), spending on average a total of 3 hours and 4 min on the Moodbuster website (SD 68, range 86-272 min) and with a total average usage duration of 5.2 weeks (SD 2.3, range 2.2-9.4).

##### Mainly Face-to-Face

A total of 10 patients (10/200, 5%) attended 3 or more FTF sessions but did not start >3 modules (3/10) or spent <1 hour on the Web-based platform (7/10). The average number of FTF sessions in this group was 6.7 (SD 3.3, range 3-12), with a mean treatment duration of 14.5 (SD 9.7, range 3.7-28) weeks. Therapists sent on average 3.6 (SD 4, range 0-12) Web-based messages. Patients started 2.5 (SD 1.0) and completed 2.1 (SD 0.6) Web-based modules on average. The mean number of logins was 4.8 (SD 1.8, range 2-8), spending on average a total of 70 min on the Moodbuster website (SD 14, range 53-97), with a total average usage duration of 7.4 weeks (SD 6.8, range 0.7-23).

##### Blended Compliant

The remaining group comprised 179 patients (179/200, 89.5%) who received ≥3 FTF sessions and started ≥3 Web-based modules while spending >1 hour on the Moodbuster website. As these patients integrated both a considerable and comparable amount on FTF and Web-based activities, they were classified as compliant with the blended treatment approach. The average number of FTF sessions was 6.5 (SD 3.0, range 3-32), with a mean treatment duration of 12.8 (SD 7.1, range 2.6-61) weeks. This was paired with an average of 10.2 (SD 6.7, range 0-39) Web-based messages from the therapist. Patients started 6 (SD 1.4) and completed 5.6 (SD 1.7) Web-based modules on average. The mean number of logins was 16.3 (SD 9.4, range 3-66), spending on average a total of 6 hours and 1 min on the Moodbuster website (SD 241, range 69-1455 min), over a total average usage period of 13.4 weeks (SD 5.1, range 2-26.5). Table 5 presents the distribution of engagement groups per country.

**Table 5:** Engagement groups with blended cognitive behavioral therapy per country.

Engagement groups	DE^a^ (n=83), n (%)	PL^b^ (n=34), n (%)	NL^c^ (n=44), n (%)	FR^d^ (n=39), n (%)
Early dropout	0 (0)	0 (0)	2 (4)	1 (2)
Mainly Web-based	3 (3)	4 (11)	0 (0)	1 (2)
Mainly face-to-face	0 (0)	1 (2)	3 (6)	6 (15)
Blended compliant	80 (96)	29 (85)	39 (88)	31 (79)

^a^DE: Germany.

^b^PL: Poland.

^c^NL: the Netherlands.

^d^FR: France.

#### Blended Compliant Versus Blended Noncompliant

A small proportion of participants (31/231, 13.4%) never started with the allocated blended intervention, and of the remaining 200 patients, 21 (21/200, 10.5%) were eventually never exposed to the blended treatment format as intended (*early dropouts*, *mainly Web-based*, or *mainly FTF*). This leaves a total group of 52 patients for whom the intended blended treatment was not applied (*blended noncompliant*). Patient characteristics of the *blended compliant* and the *blended noncompliant* groups are presented in Table 6. An association between education level and treatment compliance was observed, with a significantly higher number of patients with postsecondary education in the *blended compliant* group (54.2%), compared with 36.5% in the *blended noncompliant* group (X^2^_2_=6.0; *P*=.048). Patients in the *blended noncompliant* group had a significantly higher average number of comorbid disorders than patients compliant with the blended treatment (*t*_229_=2.107; *P*=.03). Also, a significant association between treatment preference and treatment group was observed (X^2^_2_=13.1; *P*=.001). Almost two-thirds (60%) of patients compliant with bCBT indicated at baseline a preference for the blended approach, as opposed to 42.3% in the *blended noncompliant* group; and one-third (33%) of the patients noncompliant with bCBT had a preference for TAU versus only 12% in the blended compliant group.

**Table 6:** Patient characteristics and differences between blended compliant and blended noncompliant groups.

Patient characteristics		Blended compliant (n=179)	Blended noncompliant (n=52)	Chi-square value (df)	*t* test value (df)	*P* value
Gender (female), n (%)		115 (64.2)	31 (59.6)	0.4 (1)	—^a^	.54
Age (years), mean (SD)		41.8 (12.5)	42.6 (14.2)	—	0.39 (229)	.69
**Education level, n (%)**						
	Low	18 (10.1)	10 (19.2)	6.1 (2)	—	.048
	Middle	64 (35.8)	23 (44.2)	6.1 (2)	—	
	High	97 (54.2)	19 (36.5)	6.1 (2)	—	
In a relationship, n (%)		109 (60.9)	24 (46.2)	3.6 (1)	—	—
Baseline PHQ-9^b^, mean (SD)		16.1 (4.9)	16.1 (6)	—	−0.06 (69)	.95
Number of comorbid disorders^c^, mean (SD)		0.9 (1.1)	1.3 (1.3)	—	2.11 (229)	.03
**Treatment preference, n (%)**						
	Blended	107 (59.8)	22 (42.3)	13.1 (2)	—	.001
	TAU^d^	21 (11.7)	17 (32.7)	13.1 (2)	—	.001
	Non^e^	51 (28.5)	13 (25)	13.1 (2)	—	.001

^a^Not applicable.

^b^PHQ-9: Patient Health Questionnaire-9.

^c^Assessed with the Mini International Neuropsychiatric Interview.

^d^TAU: treatment-as-usual.

^e^Non: no treatment preference.

## Discussion

### Principal Findings

The aim of this paper was to unravel the use of a blended CBT intervention for adult depression as applied in routine mental health care settings in 4 European countries. To the best of our knowledge, this is one of the first explorative studies opening the black box of blended treatment usage. We put a magnifying glass on bCBT and described in detail the usage of the blended components separately. Including log file data allowed us to examine objectively, and at a microscopic level, what actually happened during the Web-based part of the therapy. Furthermore, we aimed to reflect on the actual engagement with bCBT as compared with the intended application of the blended treatment protocol in each country, compare the application of bCBT between primary and specialized care settings, and explore general engagement with the bCBT components to identify who complies with a blended treatment approach.

Overall, patients demonstrated a large variability in the usage of the blended treatment. As typical patients do not exist in routine care, protocols may never be followed exactly as intended. The flexibility of the Web-based platform (Moodbuster) offered the option to tailor treatment to the individual patient, and as the results show, customization did indeed take place. Within the indicated guidelines of blending FTF and Web-based components, therapists and patients together created a more personalized blended care approach. This is in line with previous findings indicating that blended treatment is not a *fixed formula* and a tailored treatment plan combining the treatment modalities should be reached depending on patients’ needs, abilities, and preferences [19,21,35,49]. That patients’ treatment preferences should be taken into account in the choice of blended care is also stressed in our study by the association between baseline treatment preference and engagement with bCBT. The group of patients compliant with the bCBT format had a greater preference for the blended treatment, whereas those who did not start with the allocated bCBT indicated a preference for TAU before treatment allocation. This all can be seen as pointing in the direction of the delivery of patient-centered care and collaboration between health care provider and patient through shared decision making [50,51].

The observed patterns of treatment duration and ratio between FTF and Web-based sessions in Germany and Poland largely corresponded with the intended application. In the Netherlands and France, the Web-based part of the provided blended treatment played a less prominent role than scheduled. The mobile app was overall actively used, with patients providing on average more than two-thirds of the expected number of mood ratings. EMA counts in Germany were higher than expected, indicating that most patients rated their mood daily and on top of the prompts, also used the *self-rate* option.

In the actual application of bCBT, the distinction between primary care (Germany and Poland) and specialized care (the Netherlands and France) was visible. As by design, treatment duration in specialized care was almost twice as long as in primary care (16.2 vs 9.5 weeks) and patients attended significantly more FTF sessions (7.5 vs 5.4 FTF sessions). Nonetheless, we tend to see smaller differences in the number of FTF sessions among sites than may have been expected based on the variances in the scheduled number. Regarding the Web-based part, the patients in primary care started and completed significantly more Web-based treatment modules compared with the patients in specialized care settings; they spent on average more time on the Moodbuster website and exchanged more Web-based messages with their therapist. This reflects a more active and intensive use of the Web-based part of bCBT by patients in primary care, although the high number of exchanged Web-based messages can mainly be attributed to Germany. It should also be taken into account that in the scheduled ratio of FTF and Web-based sessions, the Web-based part of the blended treatment had a larger share in Germany. The diversity in the usage of bCBT might also be linked to the significant difference in treatment preference between primary and specialized care settings, with Dutch and French patients being less attracted to the blended approach (47.7% and 20.5%, respectively, in favor of it) compared with the Polish and German patients (77.1% and 70.6%, respectively). It should, however, be noted that TAU in primary settings was general practitioner care, which comprises mostly medication or watchful waiting.

A key observation regarding engagement was that the vast majority of patients who started with bCBT also continued with a treatment format integrating FTF and Web-based elements (bCBT *compliant* group: 179/200, 89.5%). Besides, *drop* *out* rates of bCBT in this study (with 31/231, ie, 13.5% never starting treatment and in total, 51/231, ie, 22.5% not compliant with the allocated treatment) were in line with those observed in traditional FTF CBT, being around 17% in randomized trials [52] and 25% in nonrandomized effectiveness studies [53]. This indicates that blended treatments can be applied to patient groups being treated for depression in routine mental health care. Patients who did not comply with the allocated bCBT seemed to have significantly more comorbidity. For patients with more complex psychological problems, it could be more difficult to individually walk through the Web-based modules and because of often occurring comorbidity, and even multimorbidity, content of the Web-based treatment modules may align less with their complaints. The use of a more transdiagnostic approach may help tailor the treatment to individual needs of patients with comorbid conditions, such as anxiety disorders. Finally, more patients in the *bCBT compliant* group were highly educated compared with the blended *noncompliant* group. Individual patient support needs may thus vary based on type and severity of mental disorder, combined with patient characteristics [54].

### Limitations and Future Research

This study aimed at giving a detailed description of bCBT, considering that blended treatment in routine care is a relatively new phenomenon and insights in the actual application are lacking. Although applying a more descriptive research method has its limitations, this study contributes to the existing literature by casting light on actual bCBT engagement by patients and getting an impression of their treatment fidelity. Looking into fidelity is critical to interpretation of treatment outcomes and successful implementation [55] but is often a missing element in intervention studies. Future research should focus in more detail on factors that determine usage patterns of bCBT to optimize personalized blended treatments. In addition, blended treatment engagement could be compared with studies that have looked at self-guided or therapist-guided Web-based interventions, where often lower rates of engagement have been reported [56].

This study is only the first step in unravelling the blended treatment. Many more insights into the usage of the platform, for example, can be provided with log data, such as specific usage patterns of the Web-based treatment modules or detailed interactions with the Web-based program features. Moreover, we did not include therapist factors that might have influenced patients’ usage of bCBT and how the therapists discussed the Moodbuster treatment modules and integration of components with their patients during treatment. Fine-grained process analyses, such as the influence of therapist behaviors in written feedback [57], remain an important challenge for future research. Also, of interest is further evaluation of pretreatment attitudes and acceptance of patients and therapists toward blended treatments and their potential predictors. Positive beliefs and preferences play a crucial role in the successful dissemination of new technologies [58]. Accordingly, it should be examined how to influence overall appraisal in such a way to improve uptake and implementation of bCBT in routine care. But above all, an important next step is to investigate how the actual use of the blended treatment is associated with treatment outcomes. The effectiveness of blended treatments has so far only been evaluated by looking at the treatment as a whole. The large variations in the usage of bCBT, however, underlines the importance of considering how the (combination of) different components contribute to the effects found and identifying moderating factors. In addition, engagement with the bCBT should be compared with engagement with TAU.

### Conclusions

To further explore and improve blended treatment strategies, it is important to gain insight into how the different components of bCBT treatment protocols are used by patients and therapists. Protocols may, however, not be followed exactly as they are intended. The large variability in usage of the different blended elements also indicates that a search for the *best* integration may be the wrong line of reasoning. In addition, as health care systems differ largely across countries, there might be many possible ways of applying bCBT rather than 1 standard method. 

The fact that the vast majority of patients who once started with bCBT also continued with a treatment integrating both FTF and Web-based elements indicates that blended treatments can be applied to a group of complex patients being treated for depression in routine mental health care. The next step is to gain more insight into the clinical effectiveness and cost-effectiveness of blended treatments and increase further uptake in routine mental health care.

## Appendix

Multimedia Appendix 1 Screenshots of the Moodbuster website and mobile app.

## Abbreviations

bCBT: blended cognitive behavioral therapy
CBT: cognitive behavioral therapy
E-COMPARED: European Comparative Effectiveness Research on Internet-Based Depression Treatment
EMA: ecological momentary assessment
FTF: face-to-face
MINI: Mini International Neuropsychiatric Interview
PHQ: Patient Health Questionnaire
TAU: treatment-as-usual
