# Supplementary material for: Unraveling the Black Box: Exploring Usage Patterns of a Blended Treatment for Depression in a Multicenter Study
Source: JMIR Ment Health. 2019 Jul 25;6(7):e12707. doi: 10.2196/12707 (PMC6686640; doi:10.2196/12707)

Multimedia Appendix 1. Screenshots of the Moodbuster website and mobile application

Moodbuster patient website

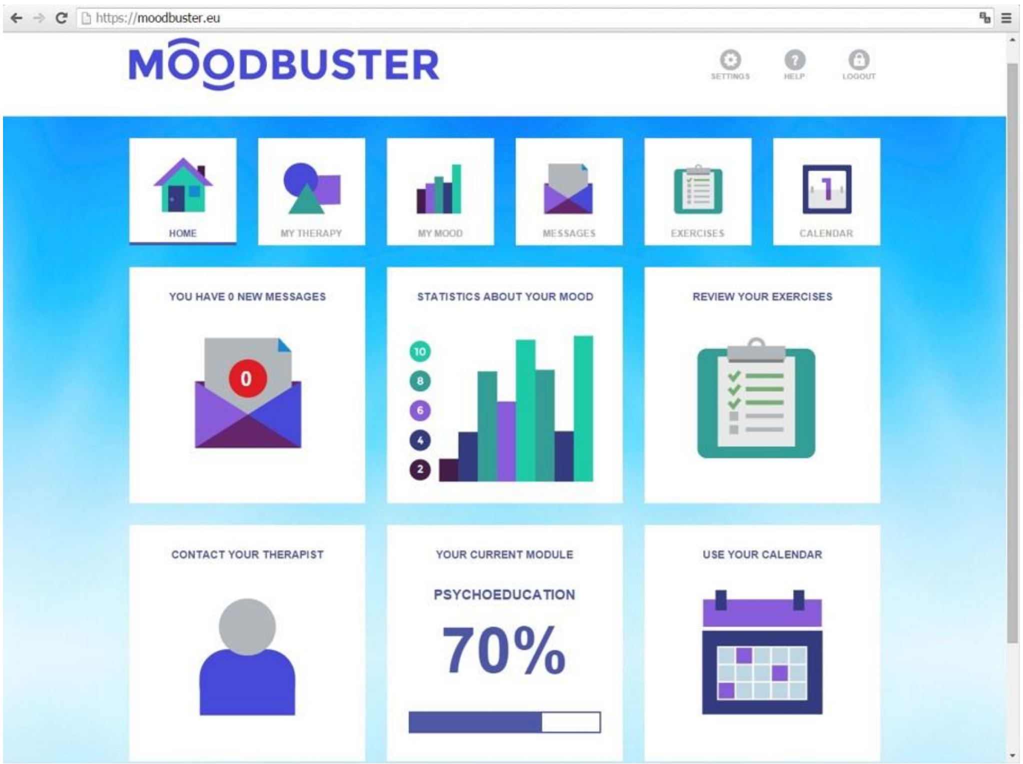

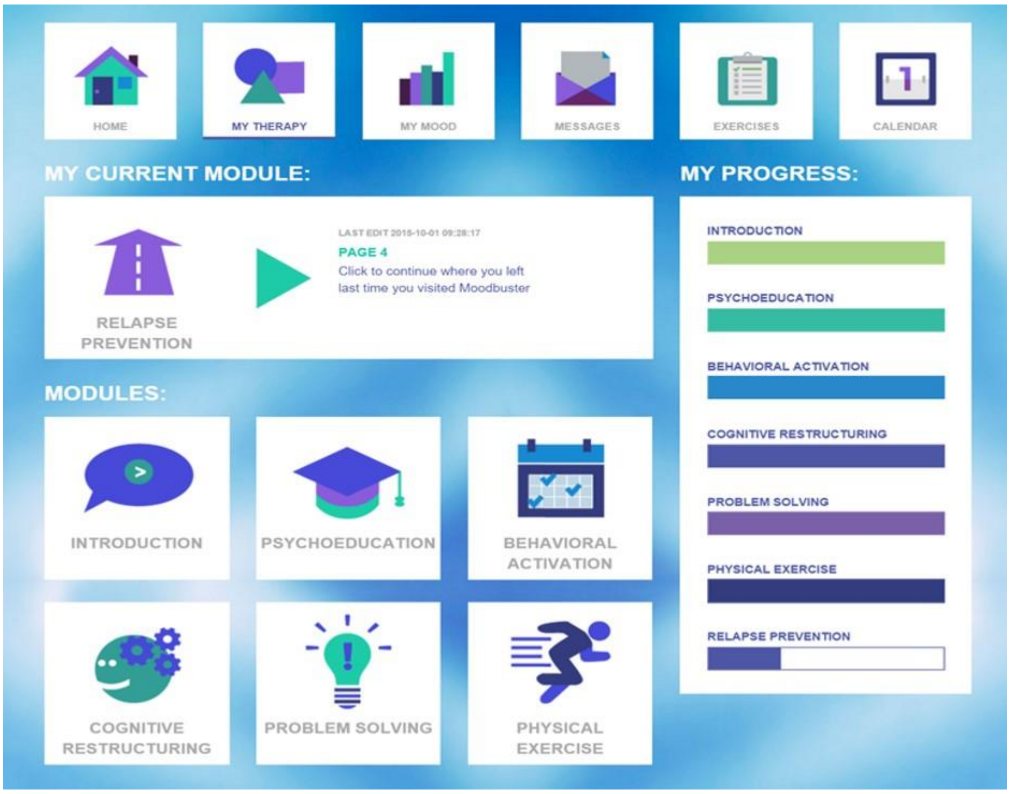

Multimedia Appendix 1. Screenshots of the Moodbuster website and mobile application

Moodbuter therapist portal

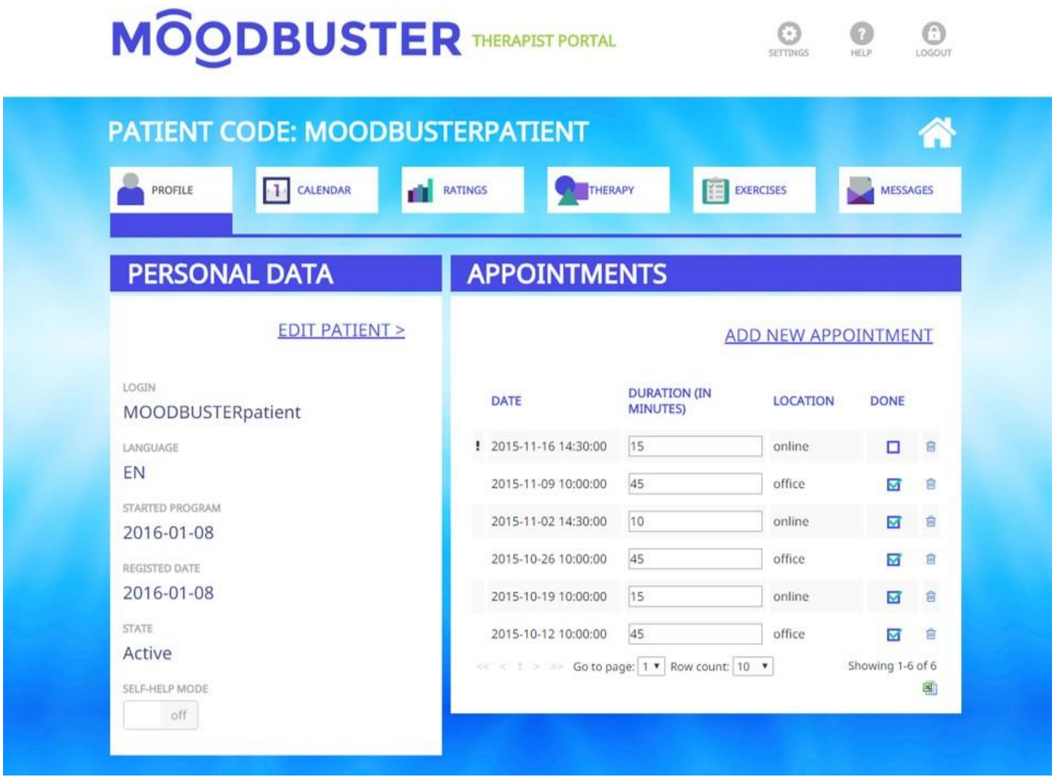

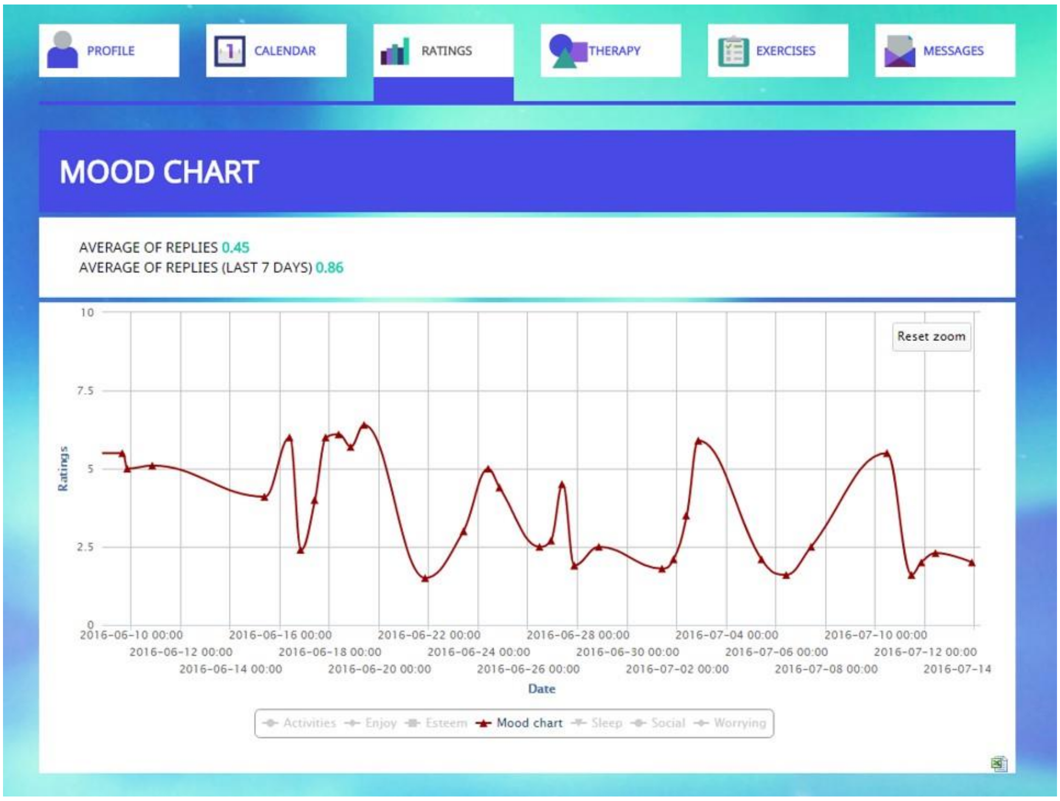

Multimedia Appendix 1. Screenshots of the Moodbuster website and mobile application

Moodbuster mobile application

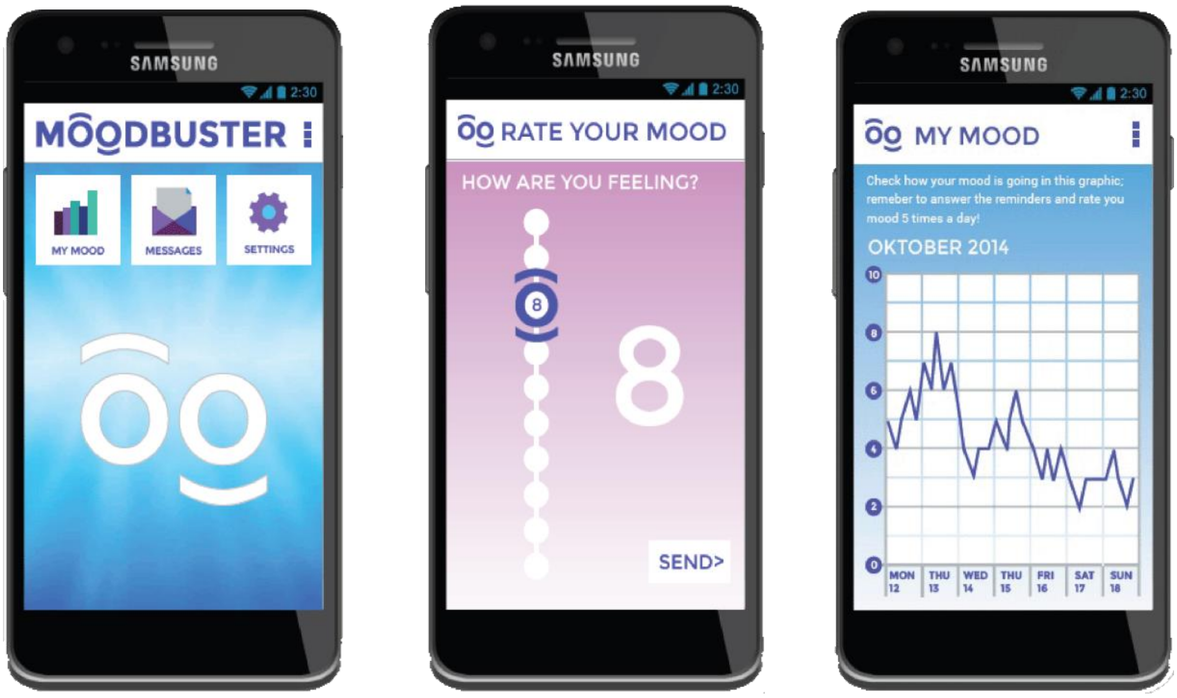

Supplement: Multimedia Appendix 1 [file mental_v6i7e12707_app1.pdf]
